# Validating the Concept of Mechanical Circulatory Support with a Rotary Blood Pump in the Inferior Vena Cava in an Ovine Fontan Model

**DOI:** 10.3390/bioengineering11060594

**Published:** 2024-06-11

**Authors:** Yves d’Udekem, Joeri Van Puyvelde, Filip Rega, Christoph Nix, Svenja Barth, Bart Meyns

**Affiliations:** 1 Department of Cardiac Surgery, The Royal Children’s Hospital, Melbourne 3052, Australia; yves.dudekem@childrensnational.org; 2 Department of Cardiac Surgery, University Hospitals Leuven, 3000 Leuven, Belgium; filip.rega@uzleuven.be (F.R.); bart.meyns@uzleuven.be (B.M.); 3 Abiomed Europe GmbH, 52074 Aachen, Germany; cnix@abiomed.com (C.N.); sbarth@abiomed.com (S.B.)

**Keywords:** Fontan, total cavopulmonary connection, cavopulmonary assist device, mechanical circulatory support, failing Fontan, cardiac assist device

## Abstract

Right-sided mechanical support of the Fontan circulation by existing devices has been compounded by the cross-sectional design of vena cava anastomosis to both pulmonary arteries. Our purpose was to investigate whether increasing inferior vena cava (IVC) flow with a rotary blood pump in the IVC only in an ovine animal model of Fontan would lead to acceptable superior vena cava (SVC) pressure. To achieve this, a Fontan circulation was established in four female sheep by anastomosing the SVC to the main pulmonary artery (MPA) and by interposing a Dacron graft between the IVC and the MPA. A rotary blood pump was then introduced in the graft, and the effect of incremental flows was observed at increasing flow regimen. Additionally, to stimulate increased pulmonary resistance, the experience was repeated in each animal with the placement of a restrictive band on the MPA distally to the SVC and Dacron graft anastomosis. Circulatory support of IVC flow alone increased the systemic cardiac output significantly, both with and without banding, indicating the feasibility of mechanical support of the Fontan circulation by increasing the flow only in the inferior vena cava. The increase in SVC pressure remained within acceptable limits, indicating the potential effectiveness of this mode of support. The findings suggest that increasing the flow only in the inferior vena cava is a feasible method for mechanical support of the Fontan circulation, potentially leading to an increase in cardiac output with acceptable increases in superior vena cava pressure.

## 1. Introduction

The long-term survival of the Fontan procedure in recent studies is excellent [1]. It is estimated that the number of people surviving with a Fontan circulation will double within the next two decades [2]. However, these patients still face a considerable burden of disease related to the physiological limitations of cavopulmonary flow, as chronic elevated systemic venous pressures and low cardiac output can alter organ perfusion, impair functionality, and eventually lead to end-organ failure [3].

Heart transplantation is not the ultimate option for all of these patients because of the sheer size of this population, the complexity related to their transplantation, and the lack of donor organs [4,5,6]. Therefore, developing a long-term mechanical support strategy, either as destination therapy or a bridge to transplantation, is necessary to support those with a failing Fontan circulation and prevent premature death. Supporting systemic circulation may not be the best option for these patients, as half of them have normal systolic function at the time of death or transplantation [7,8]. The potential for supporting the right side of the circulation has been demonstrated, but the practical long-term application of this option is precluded by the difficulty of providing an inflow from the inferior and superior vena cava and an outflow to both pulmonary arteries [9]. Most of the existing long-term assist devices provide unidirectional flow. Supporting the Fontan circulation with a rotary blood pump placed in the inferior vena cava has been achieved in a short-term animal model of Fontan but its impact on the superior vena cava pressures has not been investigated [10]. The aim of our study was to investigate if the use of a rotary blood pump in the inferior vena cava could effectively assist the Fontan circulation in an ovine Fontan animal model. Furthermore, we sought to determine whether supporting the Fontan circulation through this method would result in a reasonable increase in pressure within the superior vena cava.

## 2. Materials and Methods

### 2.1. Ethics Statement

This study was performed with the approval of and adherence to the guidelines of the animal ethics committee of the KU Leuven, according to the “Principles of Laboratory Animal Care”, formulated by the National Society for Medical Research, and in compliance with the European Commission Directive 2010/63/EU.

### 2.2. Circulatory Support

The Impella LD^®^ (Abiomed Europe GmbH, Aachen, Germany) was originally designed as a minimally invasive catheter-based cardiac assist device that can provide up to 5 L/min of blood flow from the left ventricle to the aorta. It was modified by gluing a circumferential piece of ELASTOSIL^®^ M 4601 two-component silicone rubber (Wacker Chemie AG, Munich, Germany) with an outer diameter of 12 mm to the head just distal to the inflow area. This modification was implemented to protect the pump from potential suction events that could occur around the inflow area, thereby ensuring the reliability of the device during operation.

### 2.3. Animal Model

**Figure 1:**
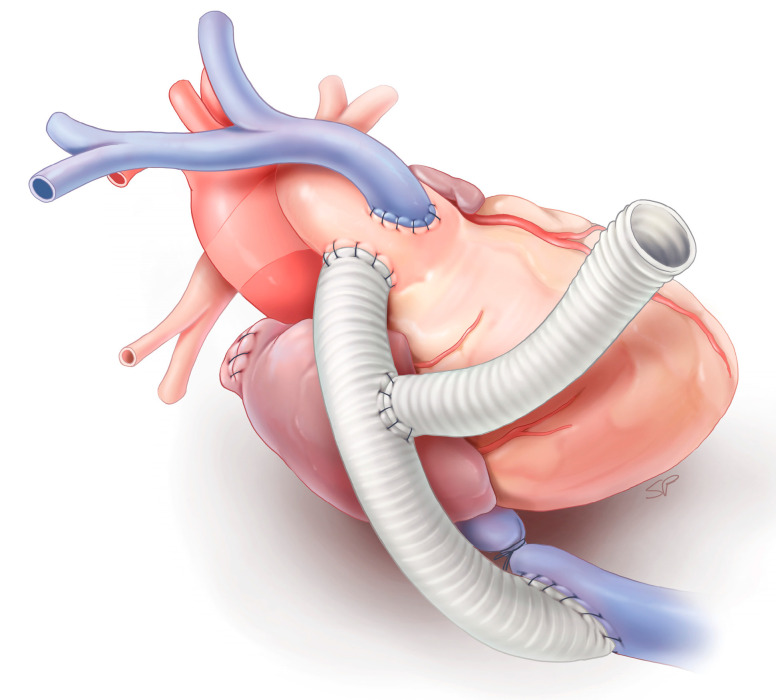
Schematic of the failing Fontan sheep model.

Four adult female sheep (Swifter × Charollais crossbreed), weighing 64.1 ± 4 kg, were obtained from the Zootechnical Center of the KU Leuven. After sedation with ketamine (4 mg/kg intravenously), anesthesia was induced (5% isoflurane inhalation), endotracheal intubation was performed, and mechanical ventilation was started. General anesthesia was maintained by isoflurane inhalation (1–3%). An arterial pressure line was placed in the left ear artery for invasive arterial pressure monitoring. A deep intravenous catheter was placed in the right jugular vein and a maintenance infusion (0.9% NaCl 20 mL/h) was started. The Fontan circulation was established in the manner previously explained [11], but with a modification in which a Dacron graft was utilized for the connection between the inferior vena cava (IVC) and the main pulmonary artery (MPA) instead of an ePTFE graft. A 14 mm Dacron graft was sutured end-to-side in the mid-portion of the IVC-MPA graft to allow for the introduction of the Impella^®^ in the section of the graft attached to the IVC (Figure 1). The side graft was then closed by using a vascular clamp to achieve occlusion. A Swan–Ganz catheter (Edwards Lifesciences, Irvine, CA, USA) was percutaneously introduced in the right internal jugular vein and the tip was advanced in the pulmonary artery bifurcation, allowing for continuous measurement of MPA and superior vena cava (SVC) pressure. A 6 Fr catheter was introduced in the left groin and the tip positioned 3–4 cm proximally to the graft–IVC anastomosis for continuous measurement of the IVC pressure. A 20 mm flow probe (Transonic Systems Inc., Ithaca, NY, USA) was placed around the aorta to continuously measure cardiac output. A 4 mm flow probe (Transonic Systems Inc., Ithaca, NY, USA) was positioned around the right carotid artery through a longitudinal, right cervical incision to continuously measure right carotid artery flow.

**Figure 2:**
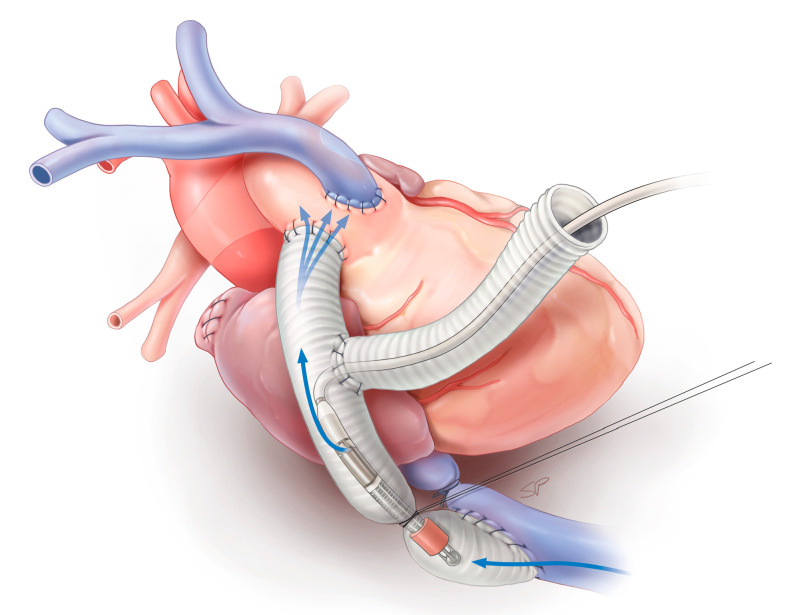
Schematic of the failing Fontan sheep model after placement of the modified Impella LD^®^ in the inferior vena cava. The blue arrows depict the flow direction from the inferior vena cava through the pump to the inferior vena cava–pulmonary artery anastomosis.

The Impella^®^ was introduced through the side graft and the tip of the pump was positioned in the graft about 2 cm above the IVC–graft anastomosis (Figure 2). Following the introduction of the device, a snare was placed around the graft and tied, thereby preventing blood loss through the side graft. A stable position of the device was ensured by fixing the catheter in the side graft. The device was started at minimal flow (10,000 rpm), mimicking no flow support, and baseline pressures and flows were recorded. A snare was tied around the IVC-MPA graft around the shaft of the Impella^®^ device to avoid recirculation. After at least two minutes of stable hemodynamics, pressure and flow measurements were taken, and the level of support was increased stepwise by 2000 rpm. This procedure was repeated until a collapse was observed in the IVC.

Secondly, an increased resistance was induced by tightening a calibrated banding around the MPA, with which approximately a 50% reduction in MPA diameter could be achieved. The banding was calibrated by tying a snare over the MPA, distal to the anastomoses, with an 18 mm Hegar dilator before removing the dilator. The device was restarted at minimal flow (10,000 rpm), with the snare untied to mimic no flow support, and pressures and flows were recorded. The snare was tied and, after at least two minutes of stable hemodynamics, pressures and flows were recorded and the level of support was increased by 2000 rpm, this process was repeated until IVC collapse. Each series of testing was repeated once before the animal was euthanized by intravenous administration of an overdose of Euthasol (Virbac USA, Fort Worth, TX, USA) after reassurance of adequate anesthesia.

### 2.4. Data Analysis and Statistics

Continuous data are presented with their mean value and standard deviation. Changes in hemodynamic status were assessed by paired Student’s *t*-test as compared with unsupported Fontan circulation. A *p*-level < 0.05 was considered significant.

## 3. Results

The Fontan circulation and assist device implantation was successful in all four animals. The animals were supported for a period of 104 ± 17.4 min before being sacrificed.

**Figure 3:**
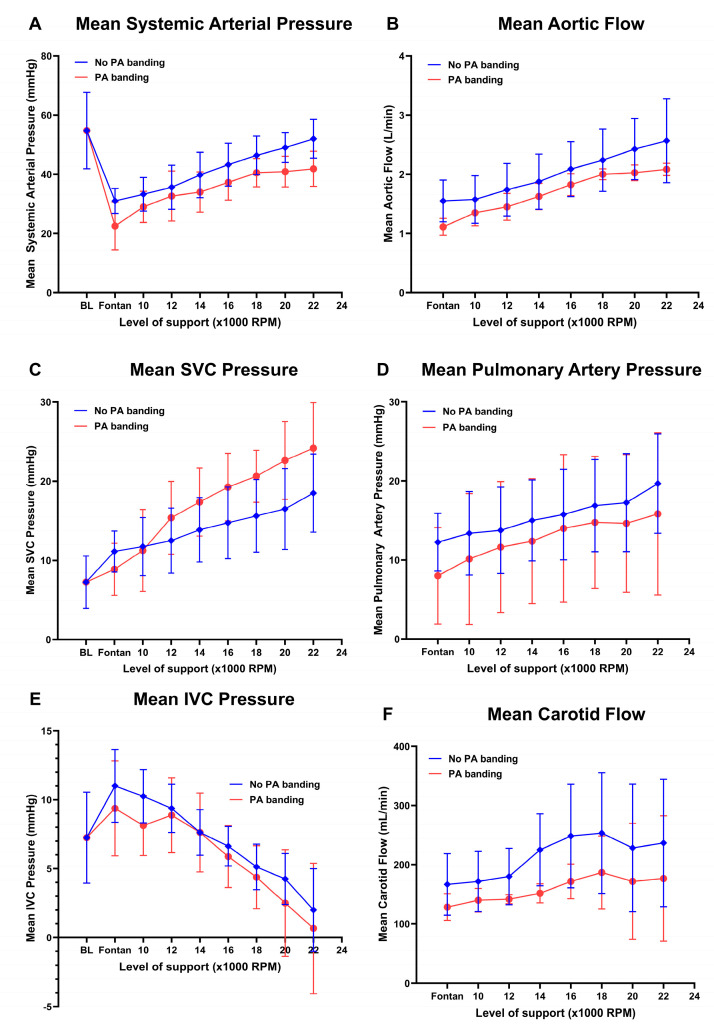
Hemodynamic data of the Fontan animals at various levels of IVC support with and without an increased afterload by banding. (**A**) Mean systemic arterial pressure. (**B**) Aortic flow. (**C**) Superior vena cava (SVC) pressure. (**D**) Mean pulmonary artery pressure. (**E**) Inferior vena cava (IVC) pressure. (**F**) Carotid flow.

The hemodynamic data of the Fontan animals at various levels of IVC support with and without an increased afterload by banding are displayed in Figure 3.

Baseline hemodynamic data of the sheep were characterized by a mean arterial pressure (MAP) of 55 ± 13 mmHg and central venous pressure of 7 ± 3 mmHg. Arterial pressure decreased significantly after establishment of the Fontan circulation to a MAP of 31 ± 4 mmHg (*p* = 0.003). Further Fontan circulation hemodynamic data of the sheep were characterized by an IVC and SVC pressure of 11 ± 3 mmHg, a mean pulmonary artery pressure of 12 ± 4 mmHg, an aortic flow of 1.6 ± 0.4 L/min, and a carotid flow of 166 ± 5 mL/min.

Circulatory support of IVC flow alone increased the systemic cardiac output from 1.6 ± 0.4 L/min to 2.6 ± 0.7 L/min (*p* = 0.039) without banding and from 1.1 ± 0.1 L/min to 2.1 ± 0.1 L/min (*p* = 0.011) with banding. On maximal flow, SVC pressure increased to a maximum of 19 ± 5 mmHg without banding (*p* = 0.017) and 24 ± 6 mmHg (*p* = 0.0289) with banding. The highest pressure reached in any animal was 30 mmHg and, provided that SVC pressure remained lower than 29 mmHg, the carotid flow remained unaffected.

Measurements were pooled starting at the first negative inflexion of the IVC pressure and the corresponding cardiac output and SVC pressure. To achieve a decrease in pressure of 5 mmHg in the IVC, a corresponding increase of 0.5 ± 0.3 L/min was necessary, leading to an increase of 3.6 ± 2.4 mmHg in pressure of the SVC.

## 4. Discussion

In this study, we successfully validated the concept of mechanical circulatory support with a rotary blood pump in the inferior vena cava in an acute ovine Fontan model.

An optimal Fontan circulation requires an unobstructed Fontan pathway, a low pulmonary vascular resistance, and a systemic ventricle with a competent atrioventricular valve, as well as an effective systolic and diastolic function [12]. Managing failing Fontan patients presents considerable challenges because the underlying causes of failure can vary, depending on which of these specific components of the Fontan circulation is affected [7,8,13,14,15]. In rare cases where systolic dysfunction is the primary mechanism of Fontan failure, the use of a systemic ventricular assist device (VAD) may be considered as a potential treatment option [16,17]. However, it is estimated that half of failing Fontan patients have normal systolic function [8]. Prêtre et al. initially demonstrated the feasibility of incorporating a paracorporeal pulsatile circulatory assist device into the subpulmonary circulation to provide mechanical support for the Fontan circulation [9]. This procedure required a complex and highly challenging surgical intervention. The difficulty of applying this concept to long-term use in patients with a Fontan circulation is related to the “cross” design of the connection between the vena cava and the pulmonary arteries. All currently available long-term VADs are designed for adults with acquired cardiomyopathy and provide unidirectional flow. The recent introduction of the EXCOR^®^ Venous Cannula has effectively streamlined and standardized the implantation process of the EXCOR^®^ circulatory assist device in the subpulmonary circulation of Fontan patients. Nevertheless, the implantation procedure still necessitates redo surgery, involving the take-down of the Fontan connections to insert the cannula [18].

Our hypothesis was that by providing mechanical support to the flow from the inferior vena cava of a Fontan circuit, we could significantly increase systemic flow with only a minimal rise in superior vena cava pressure. Our animal model appears to support this hypothesis. It was possible to increase the flow in the inferior vena cava so that systemic cardiac output almost doubled with an acceptable increase in superior vena cava pressure. However, the afterload of a patient with a failing Fontan circulation might be higher because of small or distorted central pulmonary arteries, increased resistances of the pulmonary vascular bed, or diastolic dysfunction of the systemic ventricle. Therefore, we tested this model with an imposed resistance in the form of a pulmonary artery band. Even with significant restriction to pulmonary artery blood flow, the cardiac output was significantly increased, and superior vena cave pressure increased to a level that would seem sustainable in clinical practice. It is important to note that the maximum superior vena cava pressures recorded in these conditions should be higher than those observed in an awake extubated patient, because inspiration during spontaneous ventilation is a major driving force for pulmonary blood flow. It was reassuring to note that as superior vena cava pressures increased with VAD flow regimens, no decrease in carotid blood flow was observed, provided that the generated superior vena cava pressure remained below 29 mmHg.

Several animal studies have already examined the ability of various implantable continuous flow devices to offer subpulmonary support for the Fontan circulation. Although these devices demonstrated stable hemodynamics in the short term, implementing them clinically would require aggressive surgery to modify the Fontan circulation to accommodate the devices [19,20,21,22]. Percutaneous cannulas like the Avalon elite have also been used to attempt mechanical support of the Fontan circulation during acute failure, but issues with proper placement and recirculation have limited their reliability [23]. Only one experiment has explored the feasibility of exclusively assisting the IVC using a Heartmate II in a Fontan sheep model, which resulted in an increase in aortic flow. However, the effect of IVC support on SVC pressures was not investigated [10].

### Limitations

Despite the promising results of our study, there are several limitations that need to be acknowledged. Firstly, this study was conducted using an ovine animal model, and the translation of these findings to human patients with Fontan circulation needs to be approached with caution. Additionally, the short-term nature of this study restricts our ability to draw conclusions about the long-term impact of elevated superior vena cava pressures. Ideally, a chronic animal experiment should be conducted to assess the long-term impact of elevated superior vena cava pressures. Moreover, this study’s design limited our ability to investigate potential complications resulting from the negative effect of an elevated superior vena cava pressure on the lymphatic drainage. Furthermore, this study did not address the potential complications associated with the long-term use of a circulatory assist device in the inferior vena cava. It is important to note that even with a relatively small increase in SVC pressure, the resulting pressure differential between the cavae would be substantial due to the concurrent decrease in IVC pressure. An amplified pressure differential could potentially lead to increased veno-venous collateral flow from the upper to lower body. This collateral flow bears physiological similarity to recirculation and could consequently reduce pulmonary blood flow. Although this veno-venous collateral phenomenon may not be immediately apparent in an acute model, its significance could be pronounced in a chronic model. It is also important to acknowledge that the side graft configuration in our study presents a potential risk for thrombosis due to stagnant blood flow. While no clot formation was observed in the side graft or around the device during this acute trial, it is imperative to recognize that in chronic trials, this could become an issue. In chronic animal trials or clinical applications, the device would ideally be introduced completely endovascularly, eliminating the need for a side graft with the potential for thrombus formation. Moreover, to allow for the endovascular introduction of the device, the ELASTOSIL^®^ piece and snare used in the acute trial would need to be replaced by, for instance, an inflatable cuff, which would require thorough assessment of thrombogenicity in chronic animal trials to ensure the safety and efficacy of the device in long-term applications.

## 5. Conclusions

Our animal experiment seems to demonstrate the feasibility of supporting the Fontan circulation by a circulatory assist device placed in the IVC only. Our demonstration could open the door to using an implantable long-term unidirectional flow circulatory assist device in the IVC of the Fontan circulation. There are two clinical scenarios which could benefit currently from supporting the IVC flow with a circulatory assist device. The first one is a patient with an early failure of the Fontan circulation. And the second one is the temporary support of the failing Fontan. A patient presenting with ascites, peripheral oedema, and liver failure might be supported with a circulatory assist device in the IVC as a bridge to a more conventional therapy.

In conclusion, mechanical support of the Fontan circulation by increasing the flow only in the inferior vena cava is feasible. This mode of support seems to achieve an increase in cardiac output with an acceptable increase in superior vena cava pressure.

## Data Availability

The raw data generated and analyzed during the current study are available from the corresponding author upon reasonable request.

## References

[1] d’Udekem Y., Iyengar A.J., Galati J.C., Forsdick V., Weintraub R.G., Wheaton G.R., Bullock A., Justo R.N., Grigg L.E., Sholler G.F. (2014). Redefining expectations of long-term survival after the Fontan procedure: Twenty-five years of follow-up from the entire population of Australia and New Zealand. Circulation.

[2] Schilling C., Dalziel K., Nunn R., Du Plessis K., Shi W.Y., Celermajer D., Winlaw D., Weintraub R.G., Grigg L.E., Radford D.J. (2016). The Fontan epidemic: Population projections from the Australia and New Zealand Fontan Registry. Int. J. Cardiol..

[3] Rychik J. (2016). The Relentless Effects of the Fontan Paradox. Semin. Thorac. Cardiovasc. Surg. Pediatr. Card. Surg. Annu..

[4] Kirklin J.K., Pearce F.B., Dabal R.J., Carlo W.F., Mauchley D.C. (2017). Challenges of Cardiac Transplantation Following the Fontan Procedure. World J. Pediatr. Congenit. Heart Surg..

[5] Bakhtiyar S.S., Sakowitz S., Ali K., Chervu N.L., Verma A., Si M.S., D’Alessandro D., Benharash P. (2023). Survival After Cardiac Transplantation in Adults with Single-Ventricle Congenital Heart Disease. J. Am. Coll. Cardiol..

[6] Dipchand A.I., Honjo O., Alonso-Gonzalez R., McDonald M., Roche S.L. (2022). Heart Transplant Indications, Considerations, and Outcomes in Fontan Patients: Age-Related Nuances, Transplant Listing, and Disease-Specific Indications. Can. J. Cardiol..

[7] Murtuza B., Hermuzi A., Crossland D.S., Parry G., Lord S., Hudson M., Chaudhari M.P., Haynes S., O’Sullivan J.J., Hasan A. (2017). Impact of mode of failure and end-organ dysfunction on the survival of adult Fontan patients undergoing cardiac transplantation. Eur. J. Cardiothorac. Surg..

[8] Simpson K.E., Cibulka N., Lee C.K., Huddleston C.H., Canter C.E. (2012). Failed Fontan heart transplant candidates with preserved vs impaired ventricular ejection: 2 distinct patient populations. J. Heart Lung Transplant..

[9] Prêtre R., Häussler A., Bettex D., Genoni M. (2008). Right-sided univentricular cardiac assistance in a failing Fontan circulation. Ann. Thorac. Surg..

[10] Riemer R.K., Amir G., Reichenbach S.H., Reinhartz O. (2005). Mechanical support of total cavopulmonary connection with an axial flow pump. J. Thorac. Cardiovasc. Surg..

[11] Van Puyvelde J., Rega F., Minami T., Claus P., Cools B., Gewillig M., Meyns B. (2019). Creation of the Fontan circulation in sheep: A survival model. Interact. Cardiovasc. Thorac. Surg..

[12] Gewillig M., Brown S.C. (2016). The Fontan circulation after 45 years: Update in physiology. Heart..

[13] Salaets T., Cools B., De Meester P., Heying R., Boshoff D., Eyskens B., Brown S., Meyns B., Rega F., Van Puyvelde J. (2022). Stent expansion of restrictive Fontan conduits to nominal diameter and beyond. Catheter. Cardiovasc. Interv..

[14] King G., Buratto E., Celermajer D.S., Grigg L., Alphonso N., Robertson T., Bullock A., Ayer J., Iyengar A., d’Udekem Y. (2022). Natural and Modified History of Atrioventricular Valve Regurgitation in Patients with Fontan Circulation. J. Am. Coll. Cardiol..

[15] Van Puyvelde J., Verbeken E., Gewillig M., Meyns B. (2017). Fontan failure associated with a restrictive systemic ventricle. J. Thorac. Cardiovasc. Surg..

[16] Cedars A., Kutty S., Danford D., Schumacher K., Auerbach S.R., Bearl D., Chen S., Conway J., Dykes J.C., Jaworski N. (2021). Systemic ventricular assist device support in Fontan patients: A report by ACTION. J. Heart Lung Transplant..

[17] Bedzra E.K.S., Adachi I., Peng D.M., Amdani S., Jacobs J.P., Koehl D., Cedars A., Morales D.L. (2022). Systemic ventricular assist device support of the Fontan circulation yields promising outcomes: An analysis of The Society of Thoracic Surgeons Pedimacs and Intermacs Databases. J. Thorac. Cardiovasc. Surg..

[18] Michel S.G., Menon A.K., Haas N.A., Hörer J. (2022). Cavopulmonary support with a modified cannulation technique in a failing Fontan patient. Interact. Cardiovasc. Thorac. Surg..

[19] Wei X., Sanchez P.G., Liu Y., Li T., Watkins A.C., Wu Z.J., Griffith B.P. (2015). Mechanical circulatory support of a univentricular Fontan circulation with a continuous axial-flow pump in a piglet model. ASAIO J..

[20] Derk G., Laks H., Biniwale R., Patel S., De LaCruz K., Mazor E., Williams R., Valdovinos J., Levi D.S., Reardon L. (2014). Novel techniques of mechanical circulatory support for the right heart and Fontan circulation. Int. J. Cardiol..

[21] Swartz M.F., DiVincenti L., Smith K., Westcott R., Belmont K., Harris W., Gensini F., Alfieris G.M. (2017). A modified LVAD technique to augment caval and pulmonary arterial blood flow in the “failing Fontan” circulation. J. Card. Surg..

[22] Granegger M., Escher A., Karner B., Kainz M., Schlöglhofer T., Schwingenschlögl H., Roehrich M., Podesser B.K., Kramer A., Kertzscher U. (2023). Feasibility of an Animal Model for Cavopulmonary Support with a Double-Outflow Pump. ASAIO J..

[23] Zhou C., Wang D., Condemi F., Zhao G., Topaz S., Ballard-Croft C., Zwischenberger J.B. (2019). AvalonElite Double Lumen Cannula for Total Cavopulmonary Assist in Failing Fontan Sheep Model with Valved Extracardiac Conduit. ASAIO J..

